# Human adenovirus species B knob proteins as immunogens for inducing cross-neutralizing antibody responses

**DOI:** 10.1128/msphere.00644-24

**Published:** 2024-12-13

**Authors:** Zhenwei Liu, Yuting Xian, Jixian Lan, Zhichao Zhou, Xiao Li, Rong Zhou, Dehui Chen, Xingui Tian

**Affiliations:** 1 Department of Pediatrics, The First Affiliated Hospital of Guangzhou Medical University, Guangzhou Medical University, Guangzhou, China; 2 State Key Laboratory of Respiratory Disease, National Clinical Research Center for Respiratory Disease, Guangzhou Institute of Respiratory Health, The First Affiliated Hospital of Guangzhou Medical University, Guangzhou Medical University, Guangzhou, China; 3 Guangdong Xinmai Biotechnology Co., Ltd, Guangzhou, China; 4 Deep Evolution (Guangzhou) Biotechnology Co., Ltd, Guangzhou, China; University of Michigan, Ann Arbor, Michigan, USA

**Keywords:** human adenovirus species B, knob protein, cross-neutralizing antibody response

## Abstract

The re-emerging human adenovirus (HAdV) types 3, 7, 14, and 55 of species B have caused severe or even fatal acute respiratory disease. Therefore, the development of multivalent vaccines against HAdV types 3, 7, 14, and 55 remains an important goal. In our previous study, we identified a cross-neutralizing epitope that induced broadly reactive monoclonal neutralizing antibodies against the knob proteins of HAdV types 7, 11, 14, and 55. To study the immunogenicity of HAdV species B knob proteins, we evaluated humoral immune responses to the knob proteins *in vivo* and *in vitro*. We found that the knob proteins elicited robust binding and neutralizing antibody responses after three immunizations of mice. In addition, mouse antisera raised against the knob proteins exhibited cross-neutralizing activity against original species B members. Furthermore, immunization with a mix of HAdV-3, 7, and 55 knob proteins protected Chinese tree shrews against an experimental HAdV challenge. Our results provide insight into the immunogenicity of HAdV species B knob proteins.

**IMPORTANCE:**

Human adenovirus (HAdV) species B are common pathogens causing severe pneumonia in children, and there is currently no vaccine available. Because there are many HAdV species B types, developing broad-spectrum vaccines against HAdV species B is an important research goal. Our study revealed that immunization with recombinant HAdV species B knob proteins effectively elicited cross-neutralizing antibody responses against original species B members with protective immunity. This study provides a novel insight into the immunogenicity of HAdV species B knob proteins.

## INTRODUCTION

Since human adenovirus (HAdV) was first isolated in 1953, 114 genotypes (http://hadvwg.gmu.edu/) have been identified, which are classified into seven species (from A to G) on the basis of serology and phylogenomics (1, 2). The original species B members were further classified into two subspecies, B1 (types 3, 7, 16, 21, and 50) and B2 (types 11, 14, 34, and 35). HAdV type 55 (HAdV-55), which was categorized as subspecies B2, was the product of a recombination event between HAdV-11 and HAdV-14 (3). The original B1 viruses are associated with respiratory tract infections, while the original B2 viruses, except for HAdV-14, are associated with kidney and urinary tract infections (4). Infection with HAdV-14 commonly causes acute respiratory disease (ARD). Of the HAdV species B types, HAdV-3 and HAdV-7 have been identified in epidemics globally (5–8). Numerous outbreaks of severe or even fatal ARD caused by the re-emergent HAdV-14 in species B (HAdV-B14) and HAdV-B55 have been reported in many countries over the last decade (9–11).

Vaccination is among the most effective means of preventing diseases. Currently, there is no vaccine available for use in the general population for HAdV, although an oral vaccine comprising live HAdV-E4 and B7 had been approved and used in the United States military for over 40 years (12, 13). Because there are many HAdV species B types, developing broad-spectrum vaccines against HAdV species B is an important research direction. HAdV is a non-enveloped virus with an icosahedral-shaped capsid consisting of three major proteins, the penton base, hexon, and fiber, all of which can be targeted by neutralizing antibodies (NAbs) elicited by natural infection or immunization (14). Previous studies have confirmed that hexon proteins are the major antigenic determinants recognized by NAbs against HAdV-B3, B7, B14, and B55 (15–17). Hexon protein-specific NAbs against HAdVs have serotype specificity (17, 18). Therefore, current research and development of HAdV species B multivalent vaccines involve constructing mutant hexon proteins or using a mix of hexon proteins (19–21).

Cross-reactive neutralizing humoral immunity of horse antisera raised against HAdVs was observed among original species B members (22). A previous study confirmed that fiber induced cross-NAbs to HAdV-B14 and B55 (17). In our previous study, broadly reactive monoclonal neutralizing antibodies (mNAbs) against HAdV-B7, B11, B14, and B55 were elicited by immunization with the HAdV-B11 knob protein, which was expressed in *Escherichia coli* (*E. coli*) (23). The fiber protein, which is located at the 12 vertices of the viral icosahedron, is a trimeric complex composed of an N-terminus tail domain that interacts with the penton base, a rod-like shaft domain, and a globular knob domain at the C-terminus (24). The knob domain mediates the initial interaction with attachment receptors (24). The trimeric knob domain is preferentially recognized by fiber protein-induced NAbs (14). A previous study reported that receptor-binding regions in the knob domain of HAdV-B3 elicit NAbs (25). Thus, inhibiting the virion from binding to target cells appears to be a major part of the neutralization mechanism of knob domain-specific NAbs. In this study, several HAdV species B knob proteins were expressed in *E. coli*. We demonstrated for the first time that immunization with a recombinant of HAdV species B knob proteins effectively elicited cross-NAb responses against original species B members with protective immunity.

## RESULTS

### Characterization of knob proteins

**Fig 1:**
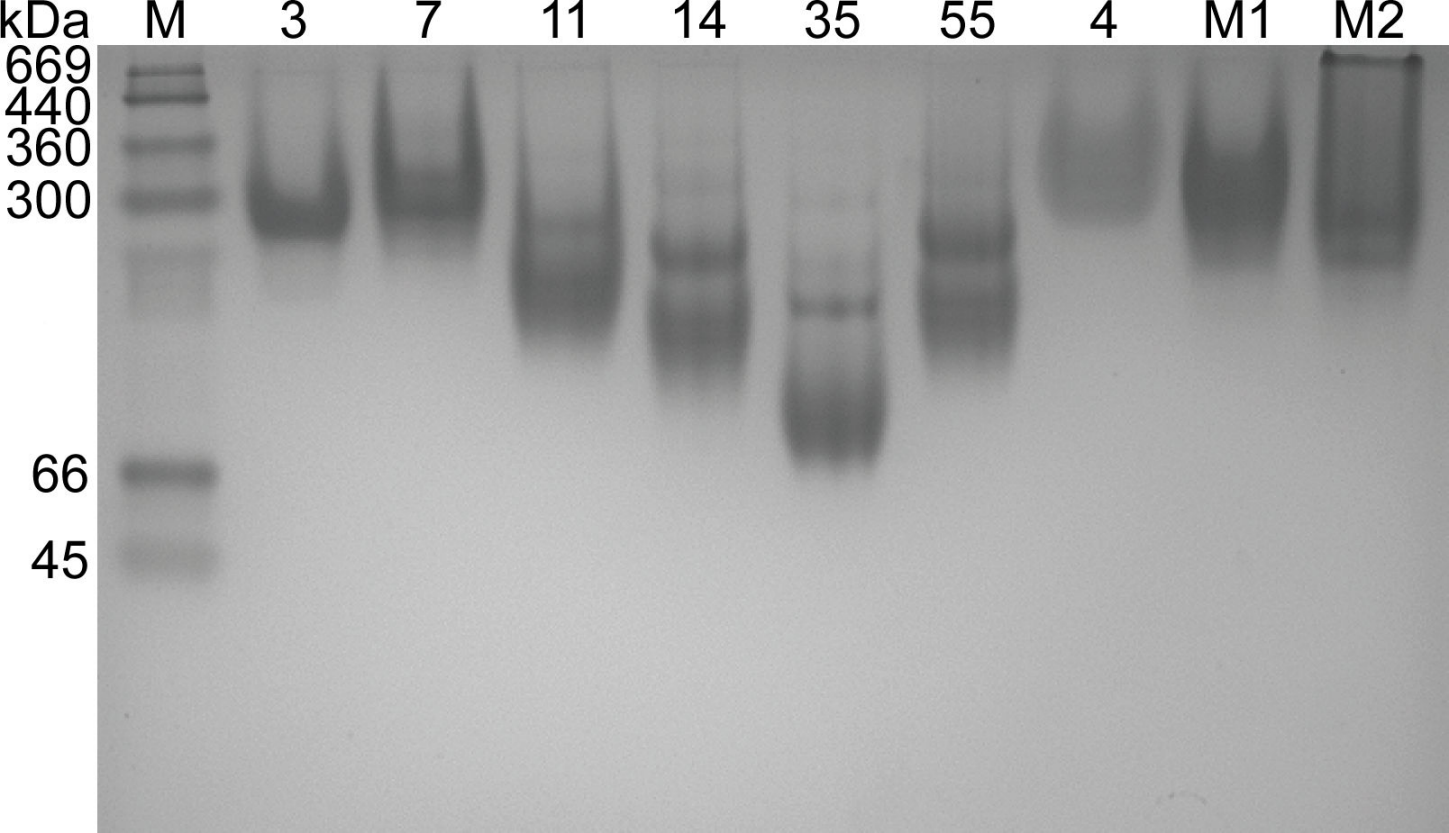
Native-polyacrylamide gel electrophoresis (native-PAGE) analysis of purified knob proteins. Purified HAdV-B3, B7, B11, B14p1, B35, B55, and E4 knob proteins and two purified mutant knob proteins, M1 and M2, were analyzed using 8% native-PAGE gel. The gel was stained with Coomassie blue. Lane M indicates the protein marker (Cat. no. MA0353-1, Meilun, Dalian, China).

Purified HAdV-B3, B7, B11, B14p1, B35, B55, and E4 knob proteins and two purified mutant knob proteins, M1 with the amino acid fragment (residues 84–143) of the HAdV-B3 knob protein replaced by that of HAdV-B7 (residues 87–148) and M2 with the amino acid fragment (residues 144–196) of the HAdV-B3 knob protein replaced by that of HAdV-B7 (residues 149–203), were analyzed by native-polyacrylamide gel electrophoresis (native-PAGE). The expected monomeric molecular weights of the purified HAdV-B3, B7, B11, B14p1, B35, B55, E4, M1, and M2 knob proteins were 23, 23, 23, 23, 28, 23, 18, 25, and 21 kDa, respectively. All the purified knob proteins are shown in polymeric form in Figure 1.

### Humoral immune responses elicited by immunization with knob proteins

**Fig 2:**
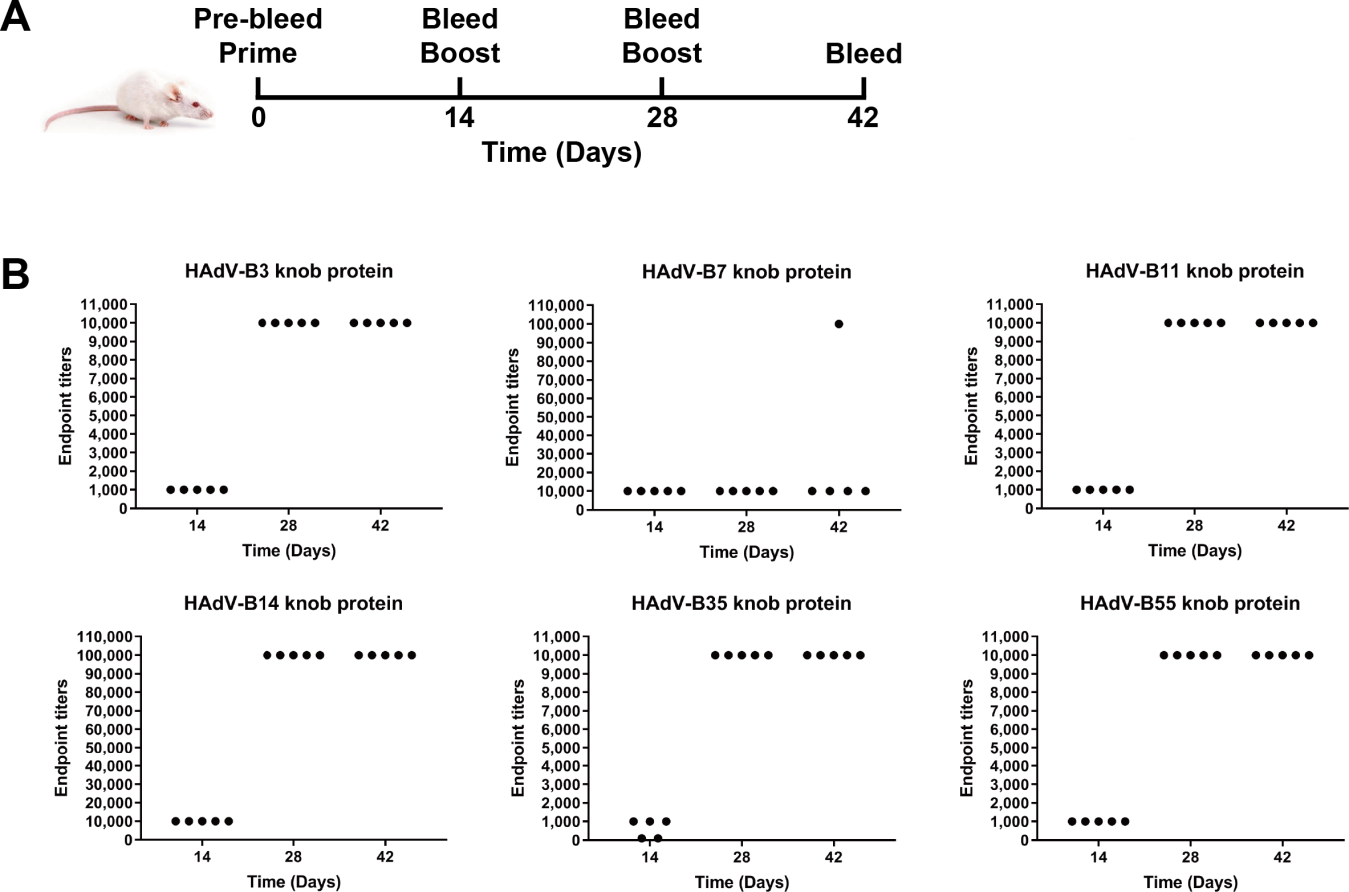
Immunogen-specific antibody responses elicited by immunization with knob proteins. (**A**) Immunization and bleed schedule. (**B**) Serum samples from different groups of mice (*n* = 5 for each group) were 10-fold serially diluted from 1:1,000 to 1:100,000, and measured for endpoint dilution titers by using the respective immunized knob protein-coated enzyme-linked immunosorbent assay on days 14, 28, and 42.

To test the immunogenicity of the knob proteins, the HAdV-B3, B7, B11, B14p1, B35, and B55 knob proteins were individually immunized in BALB/c mice on days 0, 14, and 28 (Fig. 2A). The endpoint titer of the IgG antibody in the serum from each mouse against the immunized knob proteins was determined by an enzyme-linked immunosorbent assay (ELISA) at various time points (Fig. 2A). Pre-immune sera of mice in all groups collected prior to the first immunization (day 0) were negative for the presence of IgG antibodies against the specific immunogens (titer < 1:10). The immunogen-specific antibody titers increased for all mice after the first immunization, except for two mice that had received the HAdV-B35 knob protein. The immunogen-specific antibody titers of all the mice, except for mice immunized with the HAdV-B7 knob protein, reached their peak after two immunizations, and the titers remained constant between days 28 and 42 (Fig. 2B). This suggested that the last immunization did not continue to noticeably boost the immunogen-specific antibody response. Mice in the group immunized with the HAdV-B14p1 knob protein elicited high levels of antigen-specific binding antibodies (titer = 1:100,000) compared to the other groups (Fig. 2B). Serum endpoint titers against the HAdV-B7 knob protein reached their maximum by day 14 and then remained relatively constant to the end of the experiment on day 42 (Fig. 2B). This suggested that the second and the third immunizations did not notably boost the immunogen-specific antibody response. Furthermore, antibody responses were not directed at the hexahistidine tag (His tag), as none of the sera bound to an unrelated His-tagged protein.

### Cross-NAb responses elicited by immunization with knob proteins

To determine the functional activities of the serum antibodies, a microneutralization assay (MNA) was conducted against HAdV-B3, B7, B11, B14p1, B35, B55, and E4 using sera from day 42. Sera from mice that had received phosphate-buffered saline (PBS) showed no neutralization activity (titer < 1:18) against HAdVs (Table 1). The immune sera showed the strongest neutralizing activity (titer = 1:4,608) against the virus corresponding to the knob protein (Table 1). The maximal cross-neutralizing activity was detected at a 1:4,608 dilution of the immune sera between HAdV-B14p1 and HAdV-B55 (Table 1). We speculated that this was because the HAdV-B14p1 and B55 knob proteins are similar, with a two-amino-acid deletion in HAdV-B14p1 relative to B55 (23). Sera from mice immunized with HAdV-B11 knob protein demonstrated a broad cross-neutralizing activity against HAdV-B3, B7, B14p1, and B55 with half-maximal inhibitory concentration (IC_50_) titers of 1:72, 1:18, 1:72, and 1:288, respectively (Table 1). Sera from mice immunized with HAdV-B7, B14p1, and B55 knob proteins demonstrated similar neutralization breadths for HAdV-B7, B11, B14p1, and B55. Sera from mice immunized with HAdV-B35 knob protein demonstrated a cross-neutralizing activity against HAdV-B14p1 and B55 with IC_50_ titers of 1:18 and 1:72, respectively (Table 1).

**TABLE 1:** Neutralizing antibody titers of sera from mice immunized with different knob proteins^*a*^

	HAdV-B3	HAdV-B7	HAdV-B11	HAdV-B14p1	HAdV-B35	HAdV-B55	HAdV-E4
HAdV-B3 knob protein	4,608	<18	<18	<18	<18	<18	<18
HAdV-B7 knob protein	<18	4,608	72	288	<18	288	<18
HAdV-B11 knob protein	72	18	4,608	72	<18	288	<18
HAdV-B14p1 knob protein	<18	18	18	4,608	<18	4,608	<18
HAdV-B35 knob protein	<18	<18	<18	18	4,608	72	<18
HAdV-B55 knob protein	<18	18	72	4,608	<18	4,608	<18
HAdV-E4 knob protein	<18	<18	<18	<18	<18	<18	4,608
PBS	<18	<18	<18	<18	<18	<18	<18

^*a*^ Serum samples from different groups of mice (*n* = 5 for each group) were fourfold serially diluted from 1:18 to 1:4,608, and measured for half-maximal inhibitory concentration titers using a microneutralization assay. The titer shown represents the average titer. The data are from a single experiment completed in triplicate. HAdV, human adenovirus.

### A region in HAdV-B3 knob protein elicits neutralizing activity

**Fig 3:**
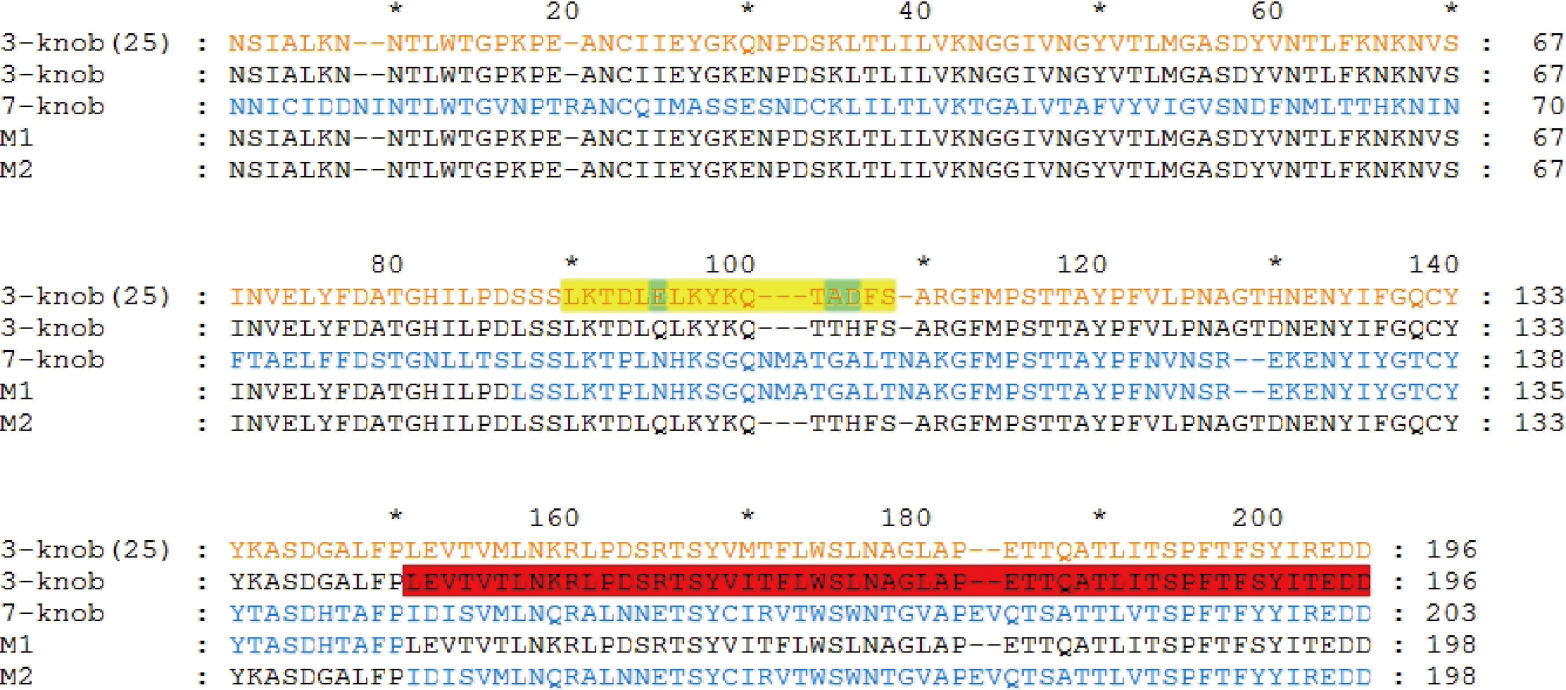
Multiple alignment of amino acid sequences of human adenovirus type 3 in species B (HAdV-B3), HAdV-B7, M1, and M2 knob proteins. The amino acid sequences of the HAdV-B3 knob protein, as described in Liebermann et al. (25), and HAdV-B3 and B7 knob proteins are colored in orange, black, and blue, respectively. The amino acid sequence of the neutralizing antigenic region is highlighted in red within the HAdV-B3 knob protein sequence. The amino acid sequence of an identified neutralizing epitope is highlighted in yellow within the HAdV-B3 knob protein sequence (25), and three key amino acids are highlighted in green.

Two mutant knob proteins, M1 and M2, were developed by separately replacing two different segments of HAdV-B3 knob protein with segments from HAdV-B7 knob protein in the corresponding position after sequence alignment (Fig. 3). Sera from mice immunized with M1 demonstrated a neutralizing activity against HAdV-B3 with an IC_50_ titer of 1:4,608 but not against HAdV-B7 (titer < 1:18), similar to that of sera from mice immunized with HAdV-B3 knob protein, while sera from mice immunized with M2 demonstrated no neutralizing activity against HAdV-B3 or B7 (Table 2). This suggested that M1 induced NAb responses against HAdV-B3, while M2 did not. This indicated that amino acids 144–196 in the C-terminal region of HAdV-B3 knob protein induced NAbs.

**TABLE 2:** Neutralizing antibody titers of sera from mice immunized with mutant knob proteins^*a*^

	HAdV-B3	HAdV-B7	HAdV-B11	HAdV-B14p1	HAdV-B35	HAdV-B55	HAdV-E4
M1	4,608	<18	<18	<18	<18	<18	<18
M2	<18	<18	<18	<18	<18	<18	<18
PBS	<18	<18	<18	<18	<18	<18	<18

^*a*^ Serum samples from different groups of mice (*n* = 5 for each group) were fourfold serially diluted from 1:18 to 1:4,608, and measured for half-maximal inhibitory concentration titers by using a microneutralization assay. The titer shown represents the average titer. The data are from a single experiment completed in triplicate. HAdV, human adenovirus.

### A knob protein mix confers protection against a viral challenge

**Fig 4:**
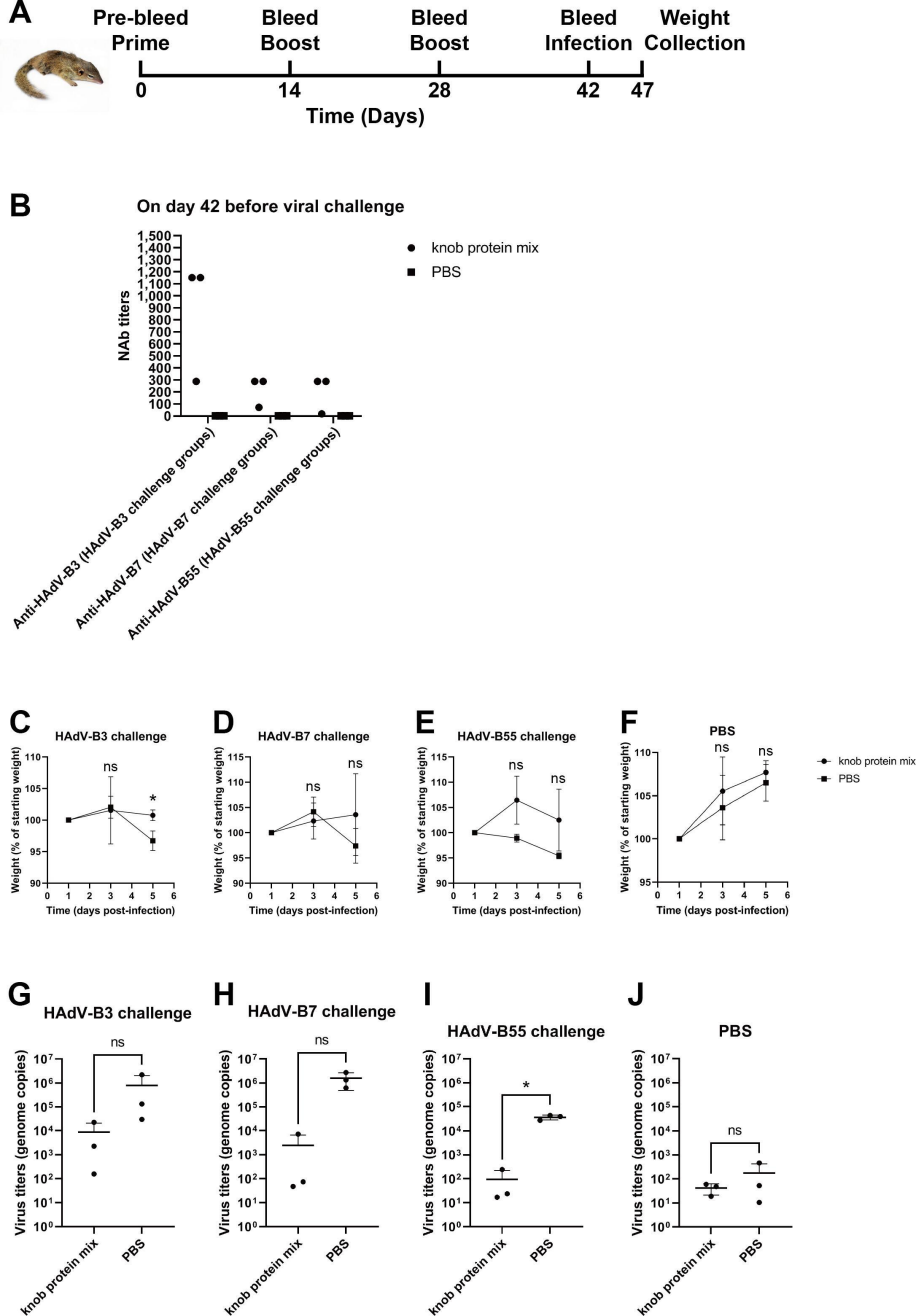
Protective efficacy conferred by the knob protein mix in Chinese tree shrews against HAdV-B3, B7, or B55 challenge. (**A**) Immunization, bleed, infection, and collection schedule. (**B**) Neutralizing antibody (NAb) IC_50_ serum titers against HAdV-B3, B7, or B55 of the experimental groups on day 42 before viral challenge. (C–F) Chinese tree shrew weight changes on days 1, 3, and 5 post-infection. (G–J) Viral DNA loads in turbinate bones on day 5 post-infection. Error bars represent mean ± standard deviation; ns, no significance; *P < 0.05.

To test if a mix of the HAdV-B3, B7, and B55 knob proteins (50 µg of each protein) conferred protection against an HAdV-B3, B7, or B55 challenge, an *in vivo* protection assay was performed using Chinese tree shrews (Fig. 4A). Following three immunizations, vaccine groups were shown to generate NAbs against HAdV-B3, B7, and B55 (as measured by an MNA) compared to the PBS control groups (Fig. 4B). Maximal neutralizing activity was detected at a 1:1,152 dilution of immune sera from two Chinese tree shrews against HAdV-B3, and the other immune serum exhibited a neutralizing activity with an IC_50_ titer of 1:288. Sera from three Chinese tree shrews demonstrated a neutralizing activity against HAdV-B7 with IC_50_ titers of 1:288, 1:288, and 1:72. Minimal neutralizing activity was detected at a 1:18 dilution of immune serum against HAdV-B55 in one Chinese tree shrew, while the other two exhibited a neutralizing activity with an IC_50_ titer of 1:288. The weight of the Chinese tree shrews was measured on days 1, 3, and 5 post-infection. The weight of the Chinese tree shrews that did not undergo a viral challenge (PBS) increased on day 3 and day 5 (Fig. 4F). The weight increased for all groups on day 3 post-infection, except for the PBS control group challenged with HAdV-B55 (Fig. 4E). The weight decreased for all groups on day 5 post-infection, except for the vaccine group challenged with HAdV-B7 (Fig. 4D). Although the weight of the Chinese tree shrews in the vaccine group was statistically different (*P* < 0.05) from that in the PBS control group on day 5 post-HAdV-B3 infection, no significant differences were observed in the weight between the vaccine groups and PBS control groups on the other days post-infection (Fig. 4C). However, the weight of all shrews in the PBS control groups with a viral challenge was less than the starting weight on day 5 post-infection but not in the vaccine groups. The mix of knob proteins-immunized group with a viral challenge exhibited a lower weight loss than the PBS control group with a viral challenge.

Next, the virus titer in turbinate bones of the Chinese tree shrews was measured, which showed that immunization with the mix of knob proteins reduced the viral load in the nose (Fig. 4G through J). However, the virus titers (genome copies) of HAdV-B55 were statistically significantly different (*P* < 0.05) only between the vaccine group and the PBS control group (Fig. 4I).

## DISCUSSION

There are two strategies for developing universal virus vaccines. One strategy involves overcoming the strain variability, such as influenza virus or human immunodeficiency virus, and the other strategy involves cross-protecting against infections with strains from the same family, such as the *Flaviviridae* Zika and dengue viruses. Previous studies had reported no cross-neutralizing activity between different HAdV species (22, 26). A previous study reported that knob protein-induced NAbs were serotype-specific in HAdV species C (27). This suggests that knob protein-induced humoral immunity to a particular serotype of HAdV species C does not provide cross-neutralization to another serotype in the same species. In this study, we found that cross-neutralizing activity occurred in sera raised by HAdV species B knob proteins. This result provides new directions for the research on multivalent vaccines against HAdV species B. In addition, all knob proteins induced potent NAb responses, and IgG titers reached their peak within two immunizations. IgG titers against the HAdV-B7 knob protein reached a maximum after a single immunization. We speculated that the HAdV-B7 knob protein may have greater immunogenicity than the other knob proteins. Therefore, HAdV species B knob proteins have potential application in the development of a multivalent subunit vaccine.

In a previous study, we identified a cross-neutralizing epitope on HAdV species B knob proteins that induced broadly reactive mNAbs against HAdV-B7, B11, B14, and B55 (23). Further analysis of the results shown in Table 1 revealed that sera from mice immunized with HAdV-B11 knob protein not only showed anti-HAdV-B7, B11, B14p1, and B55 neutralization activity but also exhibited anti-HAdV-B3 neutralization activity. Furthermore, sera from mice immunized with HAdV-B35 knob protein exhibited anti-HAdV-B14p1, B35, and B55 neutralization activity. Based on these findings, it can be inferred that in addition to the cross-neutralizing antigen epitope, we identified at least two cross-neutralizing antigen epitopes (in HAdV-B3 and B11, and in HAdV-B14p1, B35, and B55) on the original B member knob proteins that have not been identified. Furthermore, we determined that in addition to the broadly reactive mNAbs against HAdV-B7, B11, B14, and B55 that we developed, at least two broadly reactive mNAbs (one neutralizing HAdV-B3 and B11, and another neutralizing HAdV-B14p1, B35, and B5) are yet to be developed.

There are few reports identifying neutralizing epitopes on HAdV species B knob proteins. In addition to the cross-neutralizing epitope on the HAdV species B knob proteins, several linear neutralizing epitopes on the HAdV-B3 knob protein have been identified. Based on the location of the identified neutralizing epitope on the HAdV-B3 knob protein, we constructed two mutant knob proteins (M1 and M2) and found that sera from mice immunized with M1 demonstrated neutralizing activities against HAdV-B3 with a titer of 1:4,608, which was the same as that of mice immunized with HAdV-B3 knob protein. This indicated that the C-terminal region of the HAdV-B3 knob protein contains the dominant neutralization epitope. However, M2 containing the same linear neutralizing epitope of HAdV-B3 with three amino acid mutations (E92Q, A99T, and D100H) did not induce NAbs against HAdV-B3 (Fig. 3). This suggested that these three amino acids were key points for the neutralizing epitope.

The development of multivalent vaccines against HAdV-B3, B7, B14, and B55 remains an important goal. Because of the limited number of neutralizing epitopes identified in the HAdV species B knob proteins, a chimeric multivalent knob protein is not available. Therefore, to further verify the protective immunity of knob proteins *in vivo*, we prepared a mix of knob proteins. Because the HAdV-B14p1 and B55 knob proteins are similar, with a two-amino-acid deletion in the knob protein of HAdV-B14p1, we prepared a knob protein mix containing HAdV-B3, B7, and B55 knob proteins. Mice cannot support HAdV species B infection and replication, and therefore they are not suitable for evaluating HAdV species B vaccines (28). We previously reported the Chinese tree shrew as a valuable model for evaluating HAdV species B vaccines (29). Therefore, we used Chinese tree shrews to further verify the protective immunity of the knob proteins *in vivo*. Here, we showed that vaccination with the mix of knob proteins prevented weight loss after HAdV-B3, B7, and B55 challenge. HAdV species B infection led to weight loss of infected Chinese tree shrews (29). Thus, preventing weight loss is an indicator of protection against viral infection. Unexpectedly, in this study, we only found marked differences in weight between the vaccine group and the PBS control group on day 5 post-HAdV-B3 infection. We speculated that, because the average titer of NAbs against HAdV-B3 was higher than that of NAbs against HAdV-B7 and B55, this provided effective protection against the viral challenge. In addition, the viral loads in the turbinate bones of vaccinated Chinese tree shrews were reduced, which suggested that vaccination prevented or strongly reduced virus replication in the upper respiratory tract. Unexpectedly, in this study, we did not find marked differences in the protective efficacy against HAdV-B3 or B7 in turbinate bones between the vaccine group and the PBS control group. There was a significant difference in the viral loads in the turbinate bones between the vaccine group and the PBS control group following HAdV-B55 challenge. The results show that the average virus titer of HAdV-B55 was lower than that of HAdV-B3 and B7 in the PBS control groups. We speculated that the infection or replication efficiency of HAdV-B55 in the upper respiratory tract of the animals was lower than that of HAdV-B3 and B7. As a result, fewer HAdV-B55 were produced, and NAbs against HAdV-B55 were effective in neutralizing the viruses.

In summary, our study adds to our understanding of humoral immune responses to HAdV species B knob proteins *in vivo* and *in vitro*. Our data suggest the possibility of developing knob protein-based multivalent subunit vaccines against HAdV species B for humans. Future work to map the epitopes will facilitate the construction of more desirable epitope-modified recombinant knob proteins.

## MATERIALS AND METHODS

### Animals, cell, and viruses

Specific pathogen-free (SPF) 4-week-old female BALB/c mice were purchased from Guangdong Medical Laboratory Animal Center (Foshan, Guangdong, China) and were group-housed in SPF individually ventilated cages. Chinese tree shrews were purchased from the Experimental Animal Center of Kunming Medical University (Kunming, Yunnan, China) and were housed in separate cages. All animals were housed at a controlled room temperature (about 25°C) and humidity (50%–65%) with sterilized food and water available *ad libitum*. A549 cells were cultured in Dulbecco’s modified Eagle medium, nutrient mixture F-12 (DMEM/F-12) (Cat. no. C11330500BT, GIBCO BRL, Grand Island, NY, USA) supplemented with 10% heat-inactivated (56°C for 30 min) fetal bovine serum (Cat. no. 10270106, GIBCO BRL, Grand Island, NY, USA) and antibiotics (100 U/mL penicillin and 100 µg/mL streptomycin) (Cat. no. 15140122, GIBCO BRL, Grand Island, NY, USA) at 37°C in 5% CO_2_. HAdVs used in this study are listed in Table 3. The different HAdV types were individually cultured and titrated in A549 cells. A 50% tissue culture infectious dose (TCID_50_) of the virus was calculated using the Reed-Muench method, based on cytopathic effects (CPEs) (30). Different HAdV types were purified separately using a ViraTrap adenovirus mini purification kit (Cat. no. V1260, Biomiga, San Diego, CA, USA) according to the manufacturer’s instruction.

**TABLE 3:** HAdVs used in this study

Type	Strain	Reference
HAdV-B3	GZ01	(31)
HAdV-B7	GZ08	(32)
HAdV-B11	Slobitski	ATCC VR-12
HAdV-B14p1	GZ01	(33)
HAdV-B35	Holden	ATCC VR-718
HAdV-B55	Shanxi-Y16	(34)
HAdV-E4	GZ01	GenBank: KF006344.1

### Expression and purification of knob proteins

The codon-optimized genes of HAdV-B3, B7, B11, B14p1, B35, B55, and E4 knob proteins and of two mutant knob proteins, M1 with the amino acid fragment (residues 84–143) of the HAdV-B3 knob protein replaced by that of HAdV-B7 (residues 87–148) and M2 with the amino acid fragment (residues 142–196) of the HAdV-B3 knob protein replaced by that of HAdV-B7 (residues 147–203), were individually synthesized and subcloned into the pQE-30 vector (Qiagen, Hilden, Germany) at the *Bam* HI and *Hin*d III restriction sites. The recombinant plasmids were transformed into *E. coli* M15 separately. The expression and purification of the knob proteins were performed as previously described (35). Briefly, the knob protein was first expressed in bacteria. The bacteria were then lysed using a probe sonicator. The soluble knob protein was purified with a His-tag purification resin. The knob protein solution was then concentrated using a 30-kDa cutoff Amicon Ultra-15 centrifugal filter device (Cat. no. UFC801096, Millipore, Bedford, MA, USA) with a buffer exchange to PBS (pH 7.4) (Cat. no. C10010500BT, GIBCO BRL, Grand Island, NY, USA). The knob protein concentration was determined by measuring the absorbance at 280 nm using a Nanodrop One spectrophotometer (Thermo Fisher Scientific, Waltham, MA, USA). The knob protein was stored at 4°C.

### Native-PAGE

The purified HAdV-B3, B7, B11, B14p1, B35, B55, and E4 knob proteins and the purified mutant knob proteins, M1 and M2, were analyzed by native-PAGE. For this, an 8% native-PAGE gel was prepared using a native-PAGE preparation kit (Cat. no. C631101-0100, Sangon, Shanghai, China). An aliquot (30 µL) of each protein sample (1 mg/mL) was first mixed with 10 µL of 4× protein native-PAGE loading buffer (Cat. no. 9175, Takara, Dalian, China) and then loaded onto the gel. Electrophoresis was performed at 140 V for 60 min using 1× running buffer (diluted 10× tris-glycine native-PAGE running buffer, pH 8.8; Cat. no. C506035-0500, Sangon, Shanghai, China). Staining was performed using BeyoBlue Coomassie blue Super Fast staining solution (Cat. no. P0017F, Beyotime, Shanghai, China) for 1 h, and the gel was destained in distilled water overnight.

### Mouse immunization and serum sample collection

BALB/c mice were randomly divided into 10 groups (*n* = 5 mice/group) to receive purified HAdV-B3, B7, B11, B14p1, B35, B55, or E4, M1, and M2 knob proteins or PBS (blank control). Prior to injection, all mice were bled using a tail vein nick, and this naive serum served as the negative control for ELISA. On day 0, each mouse in the knob protein-immunized groups was injected intraperitoneally with 200 µL of a mixture containing 50 µg of knob protein in Freund’s complete adjuvant (FCA; Cat. no. F5881, Sigma, St. Louis, MO, USA) (knob protein solution: FCA volume ratio = 1:1). The mice were subsequently boosted on days 14 and 28 with the same dose as the priming immunization in Freund’s incomplete adjuvant (FIA; Cat. no. F5506, Sigma, St. Louis, MO, USA) (knob protein solution: FIA volume ratio = 1:1). The blank control group was injected with an equal volume of PBS in place of the knob protein solution. All mice were bled on days 14, 28, and 42. On day 42, blood samples were collected and centrifuged at 5,000 rpm for 5 min at 4°C, and serum samples were collected and held at −80°C until use.

### ELISA

The endpoint titer of the IgG antibodies in the individual serum sample against the respective immunogen was measured by ELISA and calculated as described below (36). For this assay, 96-well flat-bottom plates (Cat. no. 463201, Thermo Fisher Scientific, Waltham, MA, USA) were coated overnight at 4°C with 100 µL per well of the diluted native HAdV-B3, B7, B11, B14p1, B35, or B55 knob proteins in 1× ELISA coating buffer (Cat. no. C1050, Solarbio, Beijing, China) at a final concentration of 2 µg/mL. After washing with PBS, the coated plates were blocked with a blocking buffer (bovine serum albumin) (Cat. no. E661003-0200, Sangon, Shanghai, China) for 2 h at 37°C. Serum samples collected at each time point were serially diluted (1:10) starting at a 1:1,000 dilution in a general antibody dilution buffer (Cat. no. E661001-0500, Sangon, Shanghai, China). The plates were decanted, and the serially diluted serum was incubated in triplicate for 1 h at 25°C prior to washing with 1× washing buffer (diluted 20× ELISA washing buffer; Cat. no. E661005-0100, Sangon, Shanghai, China). Following six washes with 1× washing buffer, a 1:10,000 dilution of *ProteinFind* goat anti-mouse IgG (H+L), horseradish peroxidase conjugated (Cat. no. HS201-01, TransGen, Beijing, China), was added to the plates and allowed to incubate for 1 h at 25°C. After washing six times with 1× washing buffer, the plates were developed by adding Super TMB ELISA substrate (Cat. no. HE111-01, TransGen, Beijing, China) and quenched with an ELISA stopping solution (Cat. no. E661006-0200, Sangon, Shanghai, China), followed by colorimetric analysis at an optical density (OD) at 450 nm (OD450) and OD600 in a 96-well plate reader. Endpoint titers were determined for each serum sample on days 14, 28, and 42, and were defined as the highest reciprocal serum dilution that yielded an average absorbance (OD450 and OD600) greater than twofold that of the corresponding dilution of the blank control sera.

### MNA

To determine the cross-neutralizing activity of each serum sample against HAdV-B3, B7, B11, B14p1, B35, B55 and E4, an MNA was performed as described previously, and titers were determined (37). In brief, serial twofold dilutions of the serum (using DMEM/F-12 and beginning with a dilution of 1:9) in 96-well plates (Cat. no. 3599, Corning, Corning, NY, USA) were mixed with an equal volume of the virus stock (containing 200 TCID_50_/100 µL), and the virus-serum mixtures were incubated for 1 h at 37°C in 5% CO_2_. The serum titers ranged from 1:18 to 1:4,608. Subsequently, the virus-serum mixture was inoculated in triplicate at a volume of 100 µL per well onto A549 cell monolayers (80% confluent cultures) in the 96-well plates. After 2 h of incubation at 37°C in 5% CO_2_, the culture supernatant in each well was removed. Then, 100 µL of DMEM/F-12 was added to each well, and the cells were maintained at 37°C in 5% CO_2_. After 3 d, the cells were observed to evaluate the appearance of CPEs. Each test included control wells of uninfected and virus-only cells. The highest dilution of the sera that completely prevented CPEs in 50% of the wells was defined as the neutralization titer.

### Multiple alignment of knob protein amino acid sequences

To show the amino acid sequence of the region in which the linear neutralizing epitope of the HAdV-B3 knob protein was located, multiple alignment of the HAdV-B3 and B7, M1, and M2 knob protein amino acid sequences was performed using the ClustalX software (version 2.1) (38) with the following parameters: gap opening, 10; gap extension, 0.2; and delay divergent sequences, 30%. The Gonnet series was used for the protein weight matrix.

### *In vivo* protection assay

Chinese tree shrews were randomly divided into eight groups (*n* = 3 per group). Groups 1–4 received 150 µg of the purified HAdV-B3, B7, and B55 knob protein mix (50 µg of each protein), while groups 5–8 received PBS as a mock immunized control. On day 0, each Chinese tree shrew was injected intramuscularly with 600 µL of a mixture containing the knob protein mix or PBS with Alhydrogel adjuvant 2% aluminum hydroxide gel (Cat. no. vac-alu-250, InvivoGen, San Diego, CA, USA) (the knob protein mix solution or PBS: adjuvant volume ratio = 1:1). At an interval of 2 wk, Chinese tree shrews were boosted twice, using the same dose, adjuvant, and immunization protocol as the priming vaccination. Two weeks after the final boost immunization, the four groups that received the mix of knob proteins were challenged intranasally with 100 µL of purified HAdV-B3, B7, or B55 (1 × 10^5^ TCID_50_/100 µL for each virus) or PBS. The four groups of PBS-injected Chinese tree shrews were also challenged with the same viruses intranasally. The neutralization activity of sera against HAdV-B3, B7, or B55 in each experimental group on day 42 before the viral challenge was measured by an MNA. The weight of each Chinese tree shrew was recorded on days 1, 3, and 5 post-infection, to evaluate the protective efficacy of the knob protein mix. Turbinate bones were collected on day 5 post-infection for virus titration. HAdV genomic DNA in each tissue sample was extracted using an *EasyPure* Viral DNA/RNA kit (Cat. no. ER201-01, TransGen, Beijing, China) according to the manufacturer’s protocol and was titrated using an HAdV probe qPCR kit (Cat. no. 15-92999, TIANDZ, Beijing, China) according to the manufacturer’s protocols.

### Statistical analysis

All statistical analyses were performed with GraphPad Prism software (Version 9.5.1 [733]; GraphPad Software Inc.). *P* values < 0.05 were considered statistically significant. Comparisons of the weight changes among samples collected at various time points were evaluated by Welch’s *t*-test. Comparisons of the virus titers (genome copies) among groups were evaluated by Welch’s *t*-test.
